# SELF-AWARENESS OF SOLDIERS REGARDING RISK FACTORS FOR CARDIOVASCULAR DISEASES

**DOI:** 10.13075/ijomeh.1896.02513

**Published:** 2025

**Authors:** Magdalena Zawadzka, Aleksandra Lis, Justyna Marszalkowska-Jakubik, Pawel Szymanski

**Affiliations:** 1 Medical University of Lodz, Department of Epidemiology and Public Health, Łódź, Poland; 2 Military Institute of Hygiene and Epidemiology, Department of Organization of the Health Care System, Prevention and Treatment Team, Warsaw, Poland; 3 Medical University of Lodz, Department of Pharmaceutical Chemistry, Drug Analyses and Radiopharmacy, Łódź, Poland; 4 Military Institute of Hygiene and Epidemiology, Department of Radiobiology and Radiation Protection, Warsaw, Poland

**Keywords:** risk factors, soldiers, cardiovascular disease, military personnel, expertise, educational programs

## Abstract

**Objectives:**

Cardiovascular diseases (CVDs) remain the leading cause of death in Europe. There are more and more young people and middle-aged patients with obesity, unrecognized hypertension and metabolic abnormalities. Professional soldiers should not have CVDs. Most cardiovascular risk factors can be controlled and identified.

**Material and Methods:**

The research was conducted as the online survey. During the study, the level of knowledge regarding to cardiovascular risk factors depending on several variables, was assessed. Moreover, the assess respondents’ awareness of exposure and the level of knowledge about risk factors for CVDs and preventive measures in this area.

**Results:**

Almost one-fourth of respondents (23.4%, N = 311) indicated the knowledge of most or all cardiovascular factors such as: high level of cholesterol, tobacco smoking, advanced aged, abdominal obesity, alcohol abuse and others. The respondents demonstrated a sufficient level of factors influencing the increase of cholesterol levels in the blood.

**Conclusions:**

The results obtained during the study show that educational programs are necessary to raise awareness of cardiovascular risk factors and reduce incidence rate in the next step. Conducting training on harmful agents can result in raising general health awareness among Polish Soldiers.

## Highlights

- The prevalence of cardiovascular diseases (CVDs) increases with the age and their rank in the military corps.
- Shorter work experience and lower ranks are associated with less frequent cholesterol testing.
- A lack of knowledge about factors that increase cholesterol levels was reported by 4.1% of respondents.
- The respondents’ level of knowledge about CVD risk factors can be assessed as good.

## INTRODUCTION

Cardiovascular diseases (CVDs) are by far the leading cause of death worldwide and the most common noncommunicable diseases [1]. They are defined as a group of disorders of the heart and blood vessels. Not only do they cause premature death, but they also reduce the quality of life.

Military personnel work in particularly hazardous conditions that increase exposure to stress and directly impact mental and physical health [2]. There are data analyses concerning the correlation between the heavy responsibilities of military personnel and a greater risk of developing cardiovascular risk factors [3,4]. Specific stressors related to the military are highly correlated with acute cardiac disorders [5]. Veterans of military service may be at an even higher risk of CVDs than the general population due to more medical conditions resulting from military service. They have higher rates of mental illnesses such as depression, which are associated with increased risk factors [6]. A particular group at risk includes women after their military service. After leaving service, the risk of developing heart disease in women increases for a period of 2–5 years. In addition to traditional risk factors such as high cholesterol levels, tobacco smoking, abdominal obesity, diabetes, arterial hypertension, alcohol abuse, dietary indiscretion, lack of regular physical activity, overweight, stress, or lipid profile, mental health concerns such as PTSD and depression, and experiences of trauma, including military sexual trauma, can contribute to increased risk [7].

There are health centers focused on soldiers’ health, such as the Office of Force Resiliency. However, there is a lack of information regarding the education of soldiers in the prevention of CVDs. It seems that publicly available preventive programs could be successfully implemented in the military environment.

These programs should focus on promoting a healthy lifestyle, including increasing physical activity, improving diet, and eliminating habits such as tobacco smoking, as well as conducting regular check-ups. They should consider the specific nature of military service, including work-related stress and potential exposure to specific risk factors, as well as specific risk factors characteristic of different ethnic groups. An additional factor enhancing the attractiveness of training programs should be the use of multimedia materials, the internet, and other modern technologies. It is also necessary to consider that effective preventive programs require long-term and comprehensive actions, including changes in health and social policies.

It is estimated that up to 90% of cardiovascular disease cases can be reduced through control and management of modifiable risk factors [5]. This is why knowledge about individual risk factors and general awareness of their impact on human health are essential in disease prevention. Eliminating or limiting modifiable risk factors can reduce the risk of CVDs.

## MATERIAL AND METHODS

We would like to present the results of the research, that was carried out on a nationwide group of soldiers. The Military Center for Civic Education − Military Social Research Office was responsible for selecting the group and conducting the study. The online survey (computer-assisted web interview − CAWI) was used as a research instrument. The survey consisted of 38 substantive questions of varying degrees of complexity and 10 questions describing the respondents. The surveyed people were reached via the Military Bureau of Social Research (Wojskowe Biuro Badań Społecznych − WBBS) research panel. The study included 1331 respondents. After completing the research, WBBS provided the raw test results in the form of an Excel database.

Consent to conduct the study was obtained from the Ethics Committee (Bioethics Committee at the Military Medical Chamber in Warsaw; approval code: 11/23; approval date: May 19, 2023).

The aim of the study was to assess respondents’ awareness of exposure and the level of knowledge about risk factors for CVDs and preventive measures in this area.

The following research hypotheses were formulated:

– H0: The level of knowledge and self-awareness of soldiers regarding the risk factors of CVDs is not dependent on socio-demographic variables.
– H1: The level of knowledge and self-awareness of soldiers regarding the risk factors of CVDs depends on socio-demographic variables.

### Software and statistical analysis

The collected material was statistically processed using Excel tools and the GNU PSPP 2.0.0 program. Descriptive statistics and nonparametric data analysis methods were used. The assessment of normality distribution was performed using the Shapiro-Wilk test. The assessment of the dependence of 2 qualitative variables was performed using the Pearson χ^2^ independence test. For all analyses, the significance level was assumed at p ≤ 0.05.

### Characteristics of the respondents

Sixty out of 1331 respondents did not provide their age. In the result, the basis for calculations for this feature is 1271 respondents. Of them, 94.% were men and it was the largest group, the remaining respondents were women. A significant percentage of the respondents (46.7%) are 31–40 years old, whereas 40.8% are 41–50 years old and 6.7% are <30 years of age. At the same time, it is noticeable that at the extreme − in the group >50 years of age just >5.9% of the respondents were recorded. The average age of the surveyed people was 40.06. The largest group (dominant) are respondents aged 35 years (6.53%).

Just over 2.0% of the respondents have basic vocational education, over half (53.55%) have secondary education, whereas higher education has 44.5% of interviewee. Of the respondents, 24.9% are professional privates, just >53.4% are non-commissioned officers, 9.3% are junior officers and 12.4% are senior officers. The respondents are people with professional experience ranging 3–40 years of service. The largest group is consisted of the respondents with 15 years of service experience (11.3% of the surveyed). The average length of service is 16.8 years. During the detailed analysis, it was observed that the dominant group of study participants (48.9%) consisted of individuals with 11–20 years of service experience. Notably, 50 respondents did not provide an answer to this question. Of those surveyed, 19.3% hold staff positions, 21.9% − command positions, 28% − specialist positions, 15.3% − other specialist positions, whereas remaining 15.2% described their positions as “other.” The largest group is consisted of respondents with 15 years of service experience (11.3% of the answerers). The average length of service is 16.8 years. During detailed analysis it was observed that the dominant group of the respondents (48.9%) are people with service experience ranging 11–20 years. Fifty of the answerers did not answer this question.

Over half of the respondents (59.4%) did not take part in foreign trips or missions, as part of their official duties; the remaining 40.6% declared that they traveled abroad. Among this part of the surveyed were: men (39.6%), aged 51–60 years (46.7%), having vocational education (53.3%), senior officers (56.9%), with serve experience between 31–40 years (54.8%).

## RESULTS

Over 85% did not report CVDs. The prevalence of CVDs increases with the age of respondents and higher military service corps. A detailed analysis of the occurrence of CVDs due to socio-demographic variables is presented in Table 1.

**Table 1:** Prevalence of cardiovascular diseases (CVD) and performing a blood test to check cholesterol level due to socio-demographic variables in 2022–2023 among 1331 soldiers, online survey, Poland

Variable	CVD occurrence [%]			χ^2^	df	p	Participants performing a blood test to check cholesterol level in the last 5 years [%]			χ^2^	df	p
	no	yes					no	yes				
		in the past	at present					<1/year	≥1/year			
Gender	85.1	2.1	12.8	3.932	2	0.140	8.2	32.4	59.4	0.933	2	0.627
male	84.7	2.1	13.3				8.1	32.2	59.7			
female	91.9	2.7	5.4				9.5	36.5	54.1			
Age	85.2	2.1	12.8	74.534	6	0.000	8.0	32.4	59.6	20.470	6	0.002
25–30 years	97.6	–	2.4				9.4	42.4	48.2			
31–40 years	99.2	1.7	6.1				10.3	33.6	56.1			
41–50 years	77.0	2.7	20.3				6.0	30.4	63.6			
51–60 years	71.2	2.7	26.0				2.7	25.3	72.0			
Military service corps	85.1	2.1	12.8	25.238	6	0.000	8.1	32.3	59.6	19.160	6	0.004
professional private	90.3	1.2	8.4				11.2	30.1	58.7			
officer												
non-commissioned	84.0	2.5	13.5				7.2	34.2	58.5			
junior	90.9	1.7	7.4				9.9	38.0	52.1			
senior	74.8	2.5	22.6				4.4	23.8	71.9			
Military service tenure	85.0	2.1	12.9	85.535	6	0.000	7.7	32.4	59.8	25.987	6	0.000
3–10 years	94.4	0.4	5.2				11.9	38.8	49.3			
11–20 years	89.4	1.9	8.7				8.3	31.0	60.6			
21–30 years	71.2	3.7	25.1				4.0	30.8	65.2			
31–40 years	72.1	3.3	24.6				3.2	27.4	69.4			
Military rank	85.0	2.1	12.9	20.035	8	0.010	8.1	32.3	59.6	3.848	8	0.871
specialized (technical, equipment operation, operator, etc.)	88.2	1.9	9.9				8.5	31.5	60.0			
other specialized	88.3	2.0	9.6				7.1	35.9	57.1			
other	86.8	1.0	12.2				10.7	29.9	59.4			
staff	76.7	3.6	19.7				7.6	32.4	60.0			
other staff command (including deputy, command assistant)	84.8	1.8	13.5				7.0	32.4	60.6			

Almost 60% of respondents perform a blood test to check cholesterol levels once a year. People with shorter work experience and lower professional corps perform this type of research less often. A detailed analysis of the frequency of blood tests measuring cholesterol levels and socio-demographic variables is presented in Table 1.

The respondents demonstrated a sufficient level of knowledge influencing the increase of cholesterol levels in the blood. Of the respondents, 4.1% have no knowledge on this topic. A detailed analysis of respondents’ knowledge about factors influencing blood cholesterol levels, including socio-demographic variables, is presented in Table 2.

**Table 2:** Indication of the selected factor responsible for increased blood cholesterol levels depending on the professional military service corps, online survey among soldiers (N = 1331) in 2022–2023, Poland

Factor	Military service corps soldiers [%]					χ^2^	df	p
	professional private	non-commissioned officer	junior officer	senior officer	total			
Alcohol						0.076	3	0.995
not indicated	33.2	33.5	33.1	34.4	33.5			
indicated	66.8	66.5	66.9	65.6	66.5			
Saturated fat						5.214	3	0.157
not indicated	29.5	34.7	26.4	30.0	32.0			
indicated	70.5	65.3	73.6	70.0	68.0			
Trans fats						12.246	3	0.007
not indicated	62.1	59.7	44.6	55.6	58.4			
indicated	37.9	40.3	55.4	44.4	41.6			
Simple sugars						1.560	3	0.669
not indicated	54.3	51.4	49.6	55.0	52.4			
indicated	45.7	48.6	50.4	45.0	47.6			
Smoking						1.227	3	0.747
not indicated	55.0	52.9	51.2	50.0	52.9			
indicated	45.0	47.1	48.8	50.0	47.1			
Stress						18.001	3	0.000
not indicated	46.0	36.1	29.8	29.4	37.1			
indicated	54.0	63.9	70.2	70.6	62.9			
Obesity						7.519	3	0.057
not indicated	24.2	19.7	20.7	13.8	20.2			
indicated	75.8	80.3	79.3	86.3	79.8			
Thyroid diseases						0.835	3	0.841
not indicated	72.7	72.4	68.6	72.5	72.1			
indicated	27.3	27.6	31.4	27.5	27.9			
No opinion						2.323	3	0.508
not indicated	95.7	95.5	95.9	98.1	95.9			
indicated	4.3	4.5	4.1	1.9	4.1			

The majority of respondents assessed their level of knowledge of cardiovascular risk factors as sufficient. Every fourth respondent described it as very good, including women twice as often (47.3%) than men (22.8%). A detailed analysis in the scope is presented in Table 3.

**Table 3:** Self-assessment of respondents’ knowledge of cardiovascular disease risk factors by socio-demographic variables, online survey among soldiers (N = 1331) in 2022–2023, Poland

Variable	Participants − the level of knowledge about cardiovascular disease risk factors [%]			χ^2^	df	p
	I don't know	my knowlegde is not completed	I know most or all risk factors			
Gender	8.1	67.7	24.2	22.836	2	0.000
male	8.3	69.0	22.8			
female	5.4	47.3	47.3			
Age	8.0	68.0	24.0	13.153	6	0.041
25–30 years	8.2	68.2	23.5			
31–40 years	10.4	67.9	21.7			
41–50 years	6.0	68.2	25.8			
51–60 years	2.7	66.2	31.1			
Military service corps	8.1	67.8	24.1	14.309	6	0.026
professional private officer	10.6	68.5	20.9			
non-commissioned	8.3	68.5	23.2			
junior	6.6	64.5	28.9			
senior	3.1	65.6	31.3			
Military service tenure	7.9	67.8	24.4	10.356	6	0.110
3–10 years	9.4	64.7	25.9			
11–20 years	8.7	70.0	21.3			
21–30 years	5.5	66.2	28.3			
31–40 years	4.9	67.2	27.9			
Military rank	8.1	67.8	24.1	23.913	8	0.002
specialized (technical, equipment operation, operator, etc.)	10.4	67.6	22.0			
other specialized	4.6	64.5	31.0			
other	11.7	71.4	16.8			
staff	4.8	66.5	28.6			
other staff command (including deputy, command assistant)	7.8	69.1	23.0			

Out of 1331 respondents, 1290 answered the question, regarding knowledge connected with cardiovascular disease risk factors. Almost one-fourth of people (23.4%, N = 311) indicated the knowledge of most or all cardiovascular factors, 65.7% (N = 875) considered their level of expertise as insufficient, whereas 7.8% (N = 104) possess lack of information in this field. A detailed analysis of the data related to the respondents’ knowledge regarding cardiovascular disease risk factors is presented in Table 4.

**Table 4:** Indication of the selected risk factor for cardiovascular diseases (CVD) depending on the level of knowledge of respondents and on the military service corps, online survey among soldiers (N = 1331) in 2022–2023, Poland officertotal

Factor	Participants – the level of knowledge about (CVD) risk factors [%]				χ^2^	df	p	Military service corps soldiers [%]					χ^2^	df	p
	I don't know	my knowlegde is not completed	I know most or all risk factors	total				professional private	non-commissioned officer	junior officer	senior staff officer	total			
High levels					68.793	2	0.000						16.003	3	0.001
of cholesterol															
not indicated	36.5	12.2	5.5	12.6				17.4	12.4	9.9	5.0	12.5			
indicated	63.5	87.8	94.5	87.4				82.6	87.6	90.1	95.0	87.5			
Tobacco smoking					81.567	2	0.000						3255	3	0.354
not indicated	52.9	20.7	11.3	21.0				23.6	21.2	19.0	16.9	21.1			
indicated	47.1	79.3	88.7	79.0				76.4	78.8	81.0	83.1	78.9			
Advanced age					15.011	2	0.001						4.493	3	0.213
not indicated	74.0	65.1	55.3	63.5				62.4	64.9	55.4	65.6	63.5			
indicated	26.0	34.9	44.7	36.5				37.6	35.1	44.6	34.4	36.5			
Abdominal obesity					46.517	2	0.000						5.948	3	0.114
not indicated	52.9	28.3	18.3	27.9				28.9	29.6	22.3	21.9	27.8			
indicated	47.1	71.7	81.7	72.1				71.1	70.4	77.7	78.1	72.2			
Diabetes					36.236	2	0.000						0.709	3	0.871
not indicated	59.6	35.5	27.0	35.4				34.5	36.3	33.1	34.4	35.3			
indicated	40.4	64.5	73.0	64.6				65.5	63.7	66.9	65.6	64.7			
Arterial hypertension					15.731	2	0.000						13.128	3	0.004
not indicated	65.4	45.1	49.5	47.8				50.9	47.3	57.0	36.9	47.8			
indicated	34.6	54.9	50.5	52.2				49.1	52.7	43.0	63.1	52.2			
Alcohol abuse					3.940	2	0.139						0.968	3	0.809
not indicated	56.7	54.3	60.8	56.0				54.0	56.6	58.7	56.3	56.1			
indicated	43.3	45.7	39.2	44.0				46.0	43.4	41.3	43.7	43.9			
Dietary indiscretion					35.589	2	0.000						16.778	3	0.001
not indicated	63.5	45.6	31.8	43.7				47.2	46.1	28.1	38.8	43.8			
indicated	36.5	54.4	68.2	56.3				52.8	53.9	71.9	61.3	56.2			
Male gender					29.302	2	0.000						3.155	3	0.368
not indicated	98.1	95.3	87.1	93.6				94.1	94.2	92.6	90.6	93.6			
indicated	1.9	4.7	12.9	6.4				5.9	5.8	7.4	9.4	6.4			
Female gender					2.019	2	0.364						2.638	3	0.451
not indicated	100.0	98.7	99.4	99.0				98.4	99.0	99.2	100.0	99.0			
indicated	-	1.3	0.6	1.0				1.6	1.0	0.8	-	1.0			
Lack of regular					57.288	2	0.000						30.184	3	0.000
physical exercise															
not indicated	57.7	30.7	18.6	30.0				33.9	33.5	18.2	15.6	30.0			
indicated	42.3	69.3	81.4	70.0				66.1	66.5	81.8	84.4	70.0			
Family burden					30.638	2	0.000						14.505	3	0.002
not indicated	79.8	61.7	50.2	60.4				65.2	61.7	47.1	55.0	60.4			
indicated	20.2	38.3	49.8	39.6				34.8	38.3	52.9	45.0	39.6			
Overweight					48.333	2	0.000						9.559	3	0.023
not indicated	43.3	16.6	14.5	18.2				20.8	19.2	14.0	10.6	18.1			
indicated	56.7	83.4	85.5	81.8				79.2	80.8	86.0	89.4	81.9			
Stress					24.828	2	0.000						23.972	3	0.000
not indicated	45.2	26.6	20.3	26.6				32.3	28.3	17.4	14.4	26.6			
indicated	54.8	73.4	79.7	73.4				67.7	71.7	82.6	85.6	73.4			

Overall, respondents demonstrated a fairly good level of knowledge regarding risk factors for CVDs. Most of them correctly identified the well-known ones. However, knowledge of some risk factors for CVDs is low and insufficient. A detailed analysis of the knowledge of risk factors for CVDs depending on the professional corps of military service is presented in Table 4.

## DISCUSSION

Cardiovascular diseases remain the leading cause of death worldwide and the most common non-communicable diseases [1]. Military personnel, in turn, represent a specific occupational group with heightened exposure to stress and other CVD risk factors. They are particularly vulnerable to harmful risk factors, which makes health prevention in this group not only highly necessary but also challenging. Restrictions in food choices, high levels of stress, lack of regular exercise, and the use of substances such as cigarettes and alcohol can weaken soldiers’ physical condition and performance during exercises or missions [8,9]. The data analysis conducted in this study provides an initial understanding of the level of awareness regarding CVD risk factors among Polish soldiers and indicates directions for future research.

The authors’ study shows that every eighth respondent reported having been diagnosed with CVD in the past (13.3% of men and 5.4% of women). Studies conducted in 2008 on active military personnel, in which risk factors were measured rather than identified through self-assessment, indicated hypertension in 29.5%, hypertriglyceridemia in 24.4%, and low HDL-C levels in 20.3% of cases [10]. The authors’ study, although based on a large group of 1331 soldiers, was cross-sectional in nature and therefore limited to a questionnaire only. The authors did not examine the direct relationship between knowledge levels and the actual incidence of CVDs in this group, which would provide a more comprehensive picture of CVD risk. The significantly lower percentage compared to the aforementioned study may result from respondents’ subjective assessment. Future research should consider directly assessing disease prevalence and self-awareness of risk factors. This issue is significant and has been highlighted in the scientific literature for years. A study published in 2005 demonstrated the presence of coronary artery disease (CAD) in young Korean War combat casualties. In the analyzed group (N = 300, mean age 22.1 years), evidence of atherosclerosis was identified in 77.3% of cases, and atherosclerotic plaques were found in 65% of participants [11].

With regard to direct risk factors, a study conducted on 112 young Polish soldiers by Gielerak et al. found that cardiovascular disease risk factors were present in >50% of soldiers [10]. In the authors’ study, as many as 21% of respondents did not consider smoking to be a risk factor for CVDs. Hussain et al. [12] conducted a study on knowledge regarding the health effects of smoking among Nigerian army personnel. Only half of the respondents (50.3%) believed that smoking could limit physical fitness and military performance. Moreover, knowledge about the negative effects of smoking did not translate into their smoking behaviors [12]. This suggests that anti-smoking programs are essential among army personnel, not only in Poland. The causal impact of abnormal cholesterol levels, especially elevated LDL-C, on the development of CVDs has been unequivocally demonstrated in genetic, observational, and interventional studies. Regular monitoring of cholesterol fractions is a key element in preventing these diseases [13]. The 2002 Department of Defense survey shows that 56.3% of personnel had their cholesterol levels checked within the past 5 years [12]. The authors’ study findings align with this, with nearly 60% of respondents undergoing annual blood tests to monitor cholesterol levels. The high percentage of individuals conducting regular tests is promising; however, it should be noted that respondents had a relatively low level of knowledge regarding the risk factors responsible for elevated blood cholesterol. Research evaluating the health behaviors of Polish Army soldiers participating in the National Health Program 2016–2020 demonstrated that individuals with higher education scored significantly higher in health categories, such as proper dietary habits, preventive behaviors, and health practices, compared to respondents with secondary education. It was found that branch of service, place of residence, BMI, and level of education significantly influenced awareness of risk factors and the adoption of health-promoting behaviors [14]. In the authors’ study, we demonstrated that military rank, education level, age, and gender were correlated with the general level of knowledge about cardiovascular disease risk factors. Similarly, higher ranks and levels of education were associated with greater awareness of risk factors. However, it should be noted that more than half of the respondents (65.7%) considered their knowledge insufficient regarding cardiovascular disease risk factors.

Voluntary workplace interventions can effectively identify soldiers with unmanaged cardiovascular disease risk factors. Flynn, however, emphasizes that a more aggressive follow-up strategy is necessary to ensure that these soldiers receive the indicated medical interventions [4]. Results from a study of the U.S. Special Forces showed that good physical and mental health of soldiers was linked to their engagement in health-promoting activities. It is worth noting that participation in health-promoting behaviors is a cost-effective way to prevent diseases, such as CVDs, that are associated with an unhealthy lifestyle [15]. This undoubtedly improves job and service performance. Cardiovascular risk factors could potentially have catastrophic effects on military readiness. Systematic assessment and reinforcement of cardiovascular risk screening and risk-reduction activities should begin upon entry into service, for both active-duty soldiers and reservists, and continue throughout their military careers.

The authors’ study provides valuable insights into the level of health awareness among Polish soldiers, particularly in the area of cardiovascular disease prevention. Overall, respondents demonstrated a fairly good level of knowledge regarding risk factors for CVDs. Most of them correctly identified the well-known ones. However, knowledge of some risk factors for CVDs is low and insufficient. However, future studies should expand the scope of analysis to include direct health measurements and a more detailed correlation of sociodemographic variables with health knowledge levels.

## CONCLUSIONS

The study demonstrated that the awareness of Polish soldiers regarding specific cardiovascular risk factors is insufficient to effectively influence the formation of health-promoting habits. Individuals with shorter work experience and lower professional ranks are less likely to undergo regular blood tests for cholesterol monitoring, indicating a need for enhanced education in cardiovascular disease prevention within this group. To enhance cardiovascular disease prevention within military institutions, particularly among soldiers with shorter service and lower ranks, specific measures could be implemented, such as mandatory health training, incentive programs, informational campaigns within military units, and broad access to dietary and physiotherapy support.

Although reducing modifiable risk factors is relatively straightforward and may potentially lower the risk of CVDs, the current study did not directly assess the relationship between risk factor awareness and the actual incidence of diseases among respondents.
